# AxioSAFE: an accessible, semi-automatic filtering tool for the curation of genotyping datasets

**DOI:** 10.1093/bioadv/vbag062

**Published:** 2026-02-19

**Authors:** Lorenzo Spina, Nicholas P Howard, Stijn Vanderzande, Giorgio Tumino, Michela Troggio, Eric van de Weg, Diego Micheletti, Luca Bianco

**Affiliations:** Research and Innovation Centre, Edmund Mach Foundation, San Michele all’Adige 38098, Italy; Department of Science, Technology and Society, University School for Advanced Studies IUSS Pavia, Pavia 27100, Italy; Fresh Forward Holding Breeding & Mkt B V, Huissen 6851 ET, The Netherlands; Plant Breeding, Wageningen University and Research, Wageningen 6700AJ, The Netherlands; Plant Breeding, Wageningen University and Research, Wageningen 6700AJ, The Netherlands; Research and Innovation Centre, Edmund Mach Foundation, San Michele all’Adige 38098, Italy; Plant Breeding, Wageningen University and Research, Wageningen 6700AJ, The Netherlands; Research and Innovation Centre, Edmund Mach Foundation, San Michele all’Adige 38098, Italy; Research and Innovation Centre, Edmund Mach Foundation, San Michele all’Adige 38098, Italy

## Abstract

**Motivation:**

Genotyping datasets generated via the Thermo Fisher Axiom^®^ array are generally big, as they comprise tens of thousands of markers and hundreds of individuals, and currently, no automatic data curation pipelines are available for this kind of data. This leaves researchers with only time-consuming manual analysis as the current standard for processing these complex genotyping datasets. There is a clear need for a more efficient, streamlined approach to handle the specific quality control challenges inherent in this platform.

**Results:**

AxioSAFE (Axiom SNP Assessment and Filtering Engine) is a semi-automatic computer tool for the curation of single nucleotide polymorphism (SNP) genotyping datasets generated via Thermo Fisher Axiom^®^ array experiments. AxioSAFE provides an alternative methodology to cover a set of data curation operations, including steps such as a ploidy check, SNP filtering, Mendelian error analysis, and phasing. AxioSAFE identifies major occurrences of problematic SNPs and samples, including those not caught by the Axiom array default QC filters. Further functionality is included to let the user review identified problematic SNP classes.

**Availability and implementation:**

AxioSAFE is a Python program that can be either used via the command line interface or through a graphical user interface (GUI) and is provided as a Docker container available on DockerHub at https://hub.docker.com/r/lzspin/axiosafe, which includes all required libraries, software, and a tutorial dataset. The source code and documentation are available at https://bitbucket.org/lzspin/axiosafe/. The apple dataset used for the development of AxioSAFE is available at DOI: https://doi.org/10.5281/zenodo.18034024.

## 1 Introduction

Genomics technologies play an important role in fields that can leverage genomic information, such as plant variety selection for crop breeding. One of the most important technologies available is genotyping, which generates large datasets via sequencing technologies such as target sequencing (e.g. genotyping-by-sequencing) and genotyping arrays (Rasheed *et al.* 2017). Array technologies historically used for genotyping include Thermo Fisher Axiom^®^ arrays and Illumina Infinium^®^ microarrays. Although modern sequencing methods can address some issues of genotyping arrays, such as ascertainment bias, and are likely to be used more and more in the future, arrays offer a cheaper option that can still deliver reliable genotypes for a high number of samples and markers. Over the years, many Illumina Infinium and Thermo Fisher Axiom Single Nucleotide Polymorphism (SNP) genotyping arrays have been developed for humans and a wide array of animal and plant species, including livestock and cereal crops such as wheat and rice (Thermo Fisher 2022). More recently, arrays have also been introduced for fruit and nut crops, such as apple (Bianco *et al.* 2014, 2016, Rymenants *et al.* 2020), grapevine (Mercati *et al.* 2016), pear (Montanari *et al.* 2019), and walnut (Marrano *et al.* 2019).

The proprietary software Axiom Analysis Power Tools (APT) and Axiom Analysis Suite (AxAS) by Thermo Fisher are widely used to process raw data from Axiom arrays and return human-readable genotypic results. The “Best Practices Workflow” for Axiom data analysis includes quality control filters on samples and plates and a recurrent clustering algorithm for genotype calling of SNPs. Moreover, Axiom software can filter data based on the clustering results, in particular through SNP quality control checks that provide a first layer of data curation. However, Axiom datasets can have relatively high error rates, mainly due to the genotype calling algorithm misinterpreting certain real data cases during clustering, which may cause problems in downstream applications (Howard *et al.* 2021). These errors are not fully covered by the filters provided by the Thermo Fisher software, and the documentation does not provide a biological basis for these errors, making further analysis difficult. This also leads to the requirement of extensive post-processing for data curation. Semi-automated data curation software and methodologies are available for Illumina datasets (Di Guardo *et al.* 2015, Vanderzande *et al.* 2019), but these tools are not directly applicable to Axiom datasets due to the underlying differences in the data distribution and genotype calling algorithms, which means that current Axiom data curation involves manual and time-consuming methods. Moreover, features of genotyping datasets specific to crop species (e.g. apple’s variable ploidy and genome duplication) require dedicated solutions.

In this paper, we present AxioSAFE, a modular tool that provides an accessible pipeline to assist with data curation and streamline the analysis steps needed to generate high-quality genotyping datasets, targeting fruit crop species like apple and grapevine. Unlike tools such as PLINK (Chang *et al.* 2015) that deal with genotype calls only, AxioSAFE processes together genotype calls, signal intensity data, and clustering metrics provided by the Thermo Fisher software as output. The pipeline includes a newly developed module for ploidy assessment, new software for parentage validation and reconstruction (pedgr, https://git.wur.nl/tumin001/pedgr), and dedicated functionalities for SNP filtering that include AxAS filters and a method for the specific identification of nonstandard SNP signal patterns. Further features for fast-track SNP phasing and manual data inspection are also included, with all results collected into a persistent internal database. AxioSAFE can export the final results in standard data formats compatible with preexisting databases of curated genotyping data and other bioinformatics software (e.g. PLINK), and these outputs can act as the main source of data for enrichment of curated genotyping databases.

AxioSAFE can be used through a command line interface (CLI) or through a graphical user interface (GUI) that guides users to set up a properly formatted configuration file and to run the pipeline.

## 2 Methods

AxioSAFE is a genotyping data processing pipeline comprising several analysis steps implemented via subcommands. Three main categories of analysis can be identified for main filtering and quality control functionalities: (i) identification of non-diploid samples, (ii) identification of error-prone SNPs, and (iii) detection of parental relationships and reporting on Mendelian inconsistent errors.

**Figure 1:**
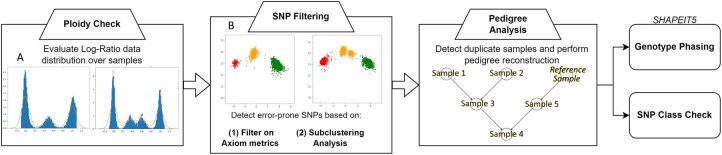
Schematic representation of AxioSAFE’s workflow. The direction represents the recommended order for each curation step category; analysis steps within a category can be skipped, or the order can be changed based on the needs of the desired application of AxioSAFE’s functionalities. (A) Frequency distribution plots: histogram representation for the frequency distribution of “Contrast” signal values, for a diploid (left) and triploid (right) sample. (B) Axiom SNP cluster plots: a standard case with three clusters (AA, AB, and BB) (left), and a problematic SNP with four clusters. For a description of the ploidy check signal distribution plots and SNP cluster plots shown here, refer to File 1, available as supplementary data at *Bioinformatics Advances* online, sections 2.2 and 3.2.

The workflow of the pipeline is reported in Fig. 1. Most commands are independent from each other, and the analyses carried out by each command can be customized through a configuration file. The full list of the AxioSAFE commands that implement the analysis, along with their parameter settings, is provided in the documentation available in AxioSAFE’s repository. The main input of AxioSAFE is the standard genotype table exported from AxAS or APT, which includes genotype calls as well as signal intensity and statistical metric data for each SNP. Instructions for exporting this file using AxAS are included in AxioSAFE’s documentation in the docs folder (see axiosafe-input-file-generation-docs.pdf). AxioSAFE is implemented in Python and is therefore cross-platform.

### 2.1 Ploidy check

The ploidy of samples is evaluated using a B-Allele Frequency (BAF)-like approach as described in Peiffer *et al.* (2006) and Chagné *et al.* (2015). In this implementation, the distribution of normalized “Contrast” values is used rather than the “Theta” values, following a fully automated procedure adapted from Howard *et al.* (2023). The number and position of the peaks in the distribution are determined using the find_peaks_cwt() function, available in the Python library SciPy (Virtanen *et al.* 2020). The resulting data are used to infer the ploidy level of each sample: a diploid sample typically shows three distribution peaks along the Y-axis, representing the two possible homozygotes (*AA* and *BB*) and the heterozygote (*AB*) genotypes. The presence of additional peaks might be indicative of polyploidy; for instance, a triploid individual generally shows four peaks, the two homozygote peaks (*AAA* and *BBB*) and the two heterozygote peaks (*AAB* and *ABB*). Samples are labeled accordingly based on the number of peaks observed. Visual examples of ploidy distributions are provided in File 1, available as supplementary data at *Bioinformatics Advances* online, section 2.2.

### 2.2 SNP filtering

The AxioSAFE SNP filters are implemented through the commands “filterm” (‘filter metrics’) and “filterc” (‘filter clusters’). These commands investigate the distributions of Axiom signal data (“Contrast” and “Size”) and genotypic calls from AxAS in the input dataset to identify problematic SNPs that may lead to Mendelian segregation errors. Initially, all SNPs are considered part of an unfiltered SNP class (‘Pass’); the filtering procedures then identify problems and assign new classes to those problematic SNPs.

AxioSAFE identifies the following main SNP classes: (i) ‘Mono High Resolution SNPs’—SNPs that display only one genotype cluster, (ii) ‘Homozygous-Homozygous Cluster SNPs’—SNPs with only two clusters that are called homozygous by AxAS, (iii) ‘Unexpectedly Distributed Cluster SNPs’- SNPs with homozygous genotype clusters having the contrast midpoint at least 1.5 from coordinate 0, (iv) ‘Axiom-metrics-threshold SNP’—SNPs flagged based on their value(s) for Axiom metrics “FLD,” “HomFLD,” “HetSO,” “HomRO,” or cluster variance or variance “z-scores,” and (v) ‘Multiple Cluster SNPs’—SNPs exhibiting more than the three Axiom standard genotype clusters.

Within each of these classes, one or more labels are used to mark SNPs in the database: labeled SNPs will be filtered out when exporting. Examples of SNP cluster plots for each of these classes and more in-depth descriptions of filtering steps are available in File 1, available as supplementary data at *Bioinformatics Advances* online, section 3.

While AxAS provides its own SNP classification method that categorizes SNPs based on the genotyping algorithm’s clustering results, AxioSAFE implements an independent filtering strategy focused on identifying problematic ‘SNPs’. This includes both filters available in AxAS and filters specific to AxioSAFE, such as the sub-clustering algorithm.

The ‘filterm’ command implements filters on the genotype counts and the metrics values available in the Axiom dataset, addressing SNP classes (i)–(iv). The ‘filterc’ command performs the sub-clustering analysis, which identifies class (v) SNPs. In this step, each of the three standard Axiom genotype clusters is analyzed independently in order to detect the presence of sub-clusters. A more detailed description of the sub-clustering approach of ‘filterc’ is available in File 1, available as supplementary data at *Bioinformatics Advances* online, section 3.5.

### 2.3 Mendelian error analysis

The AxioSAFE commands “duos” and “trios” are used to detect parent-offspring (PO) and parent-parent-offspring (PPO) relationships and to identify identical samples, by using the functions scan_duo() and scan_trio() from the pedgr R package (https://git.wur.nl/tumin001/pedgr; documentation also available in the AxioSAFE repository, “docs” folder). These steps are performed using only SNPs and individuals that passed all previous curation steps.

Both commands may accept additional input files with reference information from previously curated data (e.g. genotype data and pedigree information), which are used to enhance duplicate detection and the identification of PO and PPO relationships.

The “trios” command reports the count of Mendelian errors per SNP based on the trio relationships detected by pedgr. Based on these error reports, users may then apply the AxioSAFE “filterp” (“filter pedigree”) command to filter SNPs, excluding those with a high error count, which is implemented by assigning a separate SNP class to them, ‘Mendelian Error Filter’.

### 2.4 Phasing

The AxioSAFE command “phase” implements a fast-track feature for phasing genotypic data of SNPs passing AxioSAFE filtering steps for diploid individuals, given a marker order from an external source (i.e. the physical coordinates of the markers). Phasing is computed by the binary “phase_common” of SHAPEIT5 (Hofmeister *et al.* 2023). AxioSAFE automatically converts the unphased genotypes into the appropriate format required by the phasing algorithm, using bcftools (Danecek *et al.* 2021) for data conversion where required, and collects the results from SHAPEIT5 into variant calling format (VCF) files with phased genotypes. Optionally, the “phase” command may also use the trio relationships detected by the Mendelian error analysis commands to guide the phasing algorithm.

### 2.5 SNP class and sample review and export

AxioSAFE classifies problematic SNPs into multiple classes corresponding to different filtering criteria as described above. Each SNP class can be manually checked by using the AxioSAFE “review” command. By specifying a class, users can visually inspect the cluster plots of every SNP belonging to that class and choose via a simple user interface whether to confirm the given classification or manually filter the SNPs, excluding or including them from the final results. Samples can be manually removed using the command “review-sample.”

At the end of the pipeline, AxioSAFE can export SNPs and individuals that passed all filters (i.e. those labeled as “Pass”) to a transposed PLINK format text file (www.cog-genomics.org/plink/1.9/, (Chang *et al.* 2015), which also includes available PPO relationship information, or to a generic tabular format.

### 2.6 Implementation

AxioSAFE is implemented as a Python program and is bundled with all the needed software in a convenient Docker container. Each analysis step is executed through a command-line interface by calling the main AxioSAFE script and specifying the appropriate command for the desired analysis function. AxioSAFE uses an internal MongoDB database to collect input data and keep track of the results throughout the curation process. Final results can then be exported to a final output file using the dedicated “export” command. A configuration file supports the customization of the software by editing the parameter settings (see documentation available in AxioSAFE’s repository), while command-line parameters are primarily used for input files.

## 3 Results

The raw data used to test AxioSAFE’s performance on large datasets was the same as that used in Bianco *et al.* (2016); this dataset comes from the Apple 480K Axiom SNP genotyping array with 35-nucleotide probes for a total of 1324 samples.

A smaller subset of 187 samples (these data are available at DOI: https://doi.org/10.5281/zenodo.18034024) was selected to test all AxioSAFE steps in detail. It included two arrays, representing part of the apple diversity panel, some of which were arranged in a pedigree structure, including a subset of offspring from two full-sib families (‘Fuji’ × ‘Pinova’ and ‘Golden Delicious’ × ‘Renetta’) together with their respective parents.

An AxioSAFE workflow with default parameters and all filtering steps enabled was run to compare the pipeline with AxAS. The inputs were (i) the original, unfiltered SNP set of 487 249 SNPs and (ii) the SNP set of 389 892 ‘recommended’ SNPs reported by AxAS. AxioSAFE retained the majority of SNPs filtered by AxAS and additionally removed about 30K SNPs not excluded by AxAS, which were assigned to SNP classes ‘Mono High-Resolution SNPs’, ‘Homozygous-Homozygous Cluster SNPs’, ‘Unexpectedly Distributed Cluster SNPs’, and ‘Multiple Cluster SNPs’, yielding a subset of 366 863 SNPs. All SNP counts are reported in Table 1, available as supplementary data at *Bioinformatics Advances* online. Two out of the initial 187 samples were filtered out, as they were identified as duplicates of another sample.

The full-sib family data were then used to compute Mendelian inconsistent errors across each parent-parent-child trio for the final set of 185 samples. Mendelian error count values decreased with the filtered datasets: the unfiltered dataset (i) featured an average error count per parent-parent-offspring of 8580 (1.8%), the AxAS filtered SNPs and (ii) averaged at 2695 (0.7%) errors, while the average error count for the AxioSAFE filtered dataset further decreased to 1654 (0.45% errors per trio). Interestingly, the average error count of the 10K robust Infinium SNPs from Volk *et al.* (2022) (see Table 3, available as supplementary data at *Bioinformatics Advances* online) was 172.3 (1.7%), while it was reduced to 20.5 (0.25%) when applied to the 7946 SNPs obtained by intersecting the 366K with the 10K robust Infinium SNPs (Table 2, available as supplementary data at *Bioinformatics Advances* online).

Based on the results described above, while the current version of AxioSAFE does not remove all Mendelian inconsistent errors, it still delivers a more robust set of SNPs.

## 4 Conclusions

AxioSAFE was designed to automate genotyping data curation operations in order to obtain a robust set of phased SNPs suitable for downstream analyses. The software also identifies polyploid and duplicate samples present in the dataset, a valuable feature for diploid species that include polyploid individuals, such as apples. AxioSAFE was originally developed for plants and has been primarily tested on apples, but it has proved effective in analyzing grapevine data (Costantini *et al*. 2026, **submitted for publication**), and it is expected to be compatible with any diploid species for which an Axiom dataset is exported from AxAS or APT.

AxioSAFE currently identifies and filters a range of problematic patterns in genotyping data, at both the sample and SNP levels. However, certain aspects, such as the detection of aneuploidy, identification of sequence duplications, and automated procedures for data correction and enrichment (e.g. identification of outliers and recalling of additional alleles such as null alleles or alleles with reduced binding affinity), still need further attention. AxioSAFE was designed to be easily expanded with further functionalities thanks to its modular structure and the availability of the results through the internal database, which ensures that users can extract specific classes of SNPs and implement ad-hoc curation routines.

## Supplementary Material

vbag062_Supplementary_Data

## Data Availability

AxioSAFE is free for use and available through the GPL license. Source code and documentation are available at https://bitbucket.org/lzspin/axiosafe/. The apple dataset used for the development of AxioSAFE is available in Zenodo at DOI: https://doi.org/10.5281/zenodo.18034024.
