# Supplementary material for: AxioSAFE: an accessible, semi-automatic filtering tool for the curation of genotyping datasets
Source: Bioinform Adv. 2026 Feb 19;6(1):vbag062. doi: 10.1093/bioadv/vbag062 (PMC12967218; doi:10.1093/bioadv/vbag062)
Supplement: vbag062_Supplementary_Data [file vbag062_supplementary_data.zip › SPINA-et-al-AXIOSAFE-SUPPLEMENTARY-FILE-1-REVIEWED-VERSION.pdf]

AxioSAFE: ‘Ploidy Check’ and ‘SNP Filtering’ Rationale and Graphical Examples

AxioSAFE’s filters are designed to capture specific data patterns that can occur in Axiom datasets (cluster plots, genotype calls, the underlying clustering metrics) that are associated with inaccurate genotype calling that can lead to problems in downstream analysis. AxioSAFE filters them out to quickly generate a high quality dataset of strong SNPs and reliable samples.

Limitation: AxioSAFE performs operations that play a role in data curation; however, the pipeline targets filtering and identifying problematic SNPs and samples. AxioSAFE’s current version does not include features such as data correction and generation (e.g., genotype recalling/NA setting, calling of additional new alleles such as null alleles or alleles with reduced binding affinity, and automatic resolution of Mendel errors, e.g., via imputation). AxioSAFE’s modular structure ensures that such features may be included in future versions of the pipeline software without affecting pre-existing functionality.

(1) Preamble

Axiom Signal and True Raw Signal Data

The Axiom array platform, including the software Axiom Analysis Suite (AxAS) provides the signal data in two ways. Raw signal values extracted from the ‘CEL’ files generated by a genotyping machine are usually available as ‘summary’ data (AxAS “Summarized Signal Intensity” workflow). AxioSAFE is designed to work with the **Contrast** and **Size** signal variables (which are also called “log-ratio” and “strength”, respectively), which are the signal values used by the genotyping algorithm available in the AxAS software (“Best Practices Workflow”).

Contrast and Size are computed by the AxAS software starting from the original raw signal values loaded from the ‘CEL’ raw data files. Contrast and Size are computed by AxAS as follows:

(1) Scaling and algebraic sum: if A' and B' are the original raw signal from a ‘CEL’ file, then:
$$A = A' * 100 + 100 \text{ and } B = B' * 100 + 100$$
A and B signal intensity values are reported in the AxAS output file ‘AxiomGT1.summary.txt’, and they can be used to deduce the original values of A’ and B’.

(2) Logarithm transformation: Given *A* and *B* computed from the previous step, Contrast/Log-Ratio and Size/Strength are computed as follows:
$$Contrast = log_2\left(\frac{A}{B}\right)$$
$$Size = \frac{log_2 A + log_2 B}{2}$$

Based on the formulas, Contrast depends on which value between A and B is the higher one and by how much that is the case, while Size is a representation of the signal strength.

These transformations affect the patterns in the signal data that can be recognized across SNPs, and therefore the applicability of data curation approaches designed for other array platforms. Details and possible examples of these patterns will be covered in this document, with particular attention to SNP cluster plot patterns, but it is important to take into account that some of the variability depends on these mathematical operations, regardless of the underlying biology and technology.

(2) Ploidy Check

(2.1) Polyploidy in plant samples

Polyploidy is a known aspect of certain crop species. The current version of AxioSAFE targets species that are primarily diploid, such as apple and grapevine, where polyploidy may occur in individuals within a panel. The 'Ploidy Check' operations, implemented by the AxioSAFE `ploidy` command, examine the distribution of Axiom signal data to detect instances of polyploidy, and label the samples accordingly to exclude them from subsequent analysis steps in the pipeline.

Limitation: Currently, AxioSAFE assumes that non-diploid samples have consistent ploidy across all chromosomes. As a result, aneuploidy (i.e., variation of ploidy limited to a subset of the chromosomes in the genome) is not taken into account.

(2.2) Algorithm: Study of the Frequency Distribution of Signal Values

The AxioSAFE `ploidy` command implements a check on the ploidy of the sample using the distribution of the Axiom signal variable Contrast (i.e., 'log-ratio').

The methodology used by AxioSAFE for the ploidy identification was inspired by similar B-Allele approaches in human genetics (Peiffer *et al.*, 2006) and available in Illumina genotyping software (Bead Studio, GenomeStudio for Infinium SNP arrays), which were later introduced in plant genetics, with B-Allele Frequency (BAF) methods for polyploid and aneuploid identification (Chagné *et al.*, 2015). The usage of a BAF method for data curation in AxioSAFE is based on (Vanderzande *et al.*, 2019), while the basis of the codebase in AxioSAFE that implements the BAF method comes from the implementation of the ploidyClassifier program in (Howard *et al.*, 2023).

Due to the nature of Axiom signal data (see section "Axiom Signal Data and True Raw Signal") the values of Contrast are not consistent with Infinium datasets. For this reason, the data undergoes two separate steps of data transformation:

1) Contrast data is 'normalized'. In particular, the data is rescaled: the 'AA.meanX' and 'BB.meanX' Axiom metric values define the new '0' and '1' values of the new scale. The formula below is used: for x set of Contrast signal values, the normalized values x_{norm} is

$$x_{norm} = \frac{x - BBmeanX}{AAmeanX - BBmeanX}.$$

2) A set of data-cleaning filters is applied to SNPs, filtering out noise-heavy SNPs (usually associated with certain characteristics including: polymorphic SNPs at multiple loci, secondary polymorphisms at the probe site, SNPs with Copy Number Variations) and keeping only the ones most informative for ploidy identification.
The filters are generic and include, for each SNP:

a) Using only Poly High Resolution ConversionType category SNPs as identified by the Axiom software.
b) Filter on the minimum size of the cluster space on the x-axis (default 4, but should be changed based on the sample size).
c) Threshold on the maximum amount of missing data tolerated (default 20, but should be changed based on the sample size).
d) Threshold on the minimum distance between the heterozygous cluster and the homozygous clusters (default 0.25).

The configuration file holds parameters to customize settings for these preliminary filters.

With these preliminary steps, we obtain a distribution that we can study by using methods previously described for Infinium datasets; a comparison through frequency distribution plots for the distribution before and after the transformation is found in Figure 1.

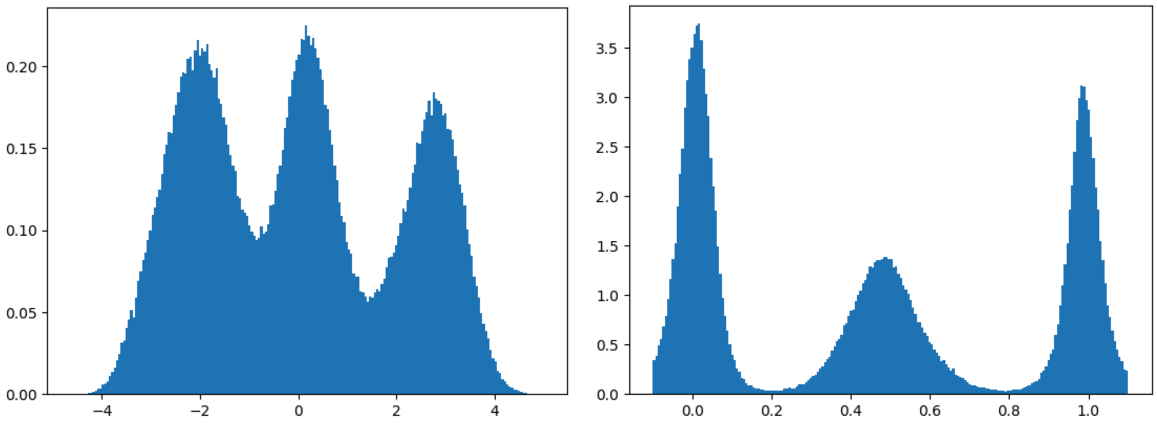

Figure 1. Frequency distribution plot; histogram representation for the frequency distribution of "Contrast" signal values for an Apple plant sample. The histogram plot on the left shows the original distribution of the Axiom signal variable 'Contrast', the right one shows the same sample after filtering SNPs and normalizing the data range (x-axis).

Once a set of SNPs with normalized Contrast data is obtained, AxioSAFE will begin the ploidy check algorithm. The main steps are:

1) Generation of frequency distribution (represented via a histogram plot visualization)
2) Application of a density function based on the given frequency distribution
3) Identification of the coordinates of the peaks from the density function, using the function `find_peaks_cwt()`.

The algorithm identifies the polyploidy of samples with a method comparable to the manual check of frequency distribution histogram plots. The frequency will naturally have areas of higher frequency of values corresponding to the coordinates where most calls for a certain genotype are found. For instance, in a diploid sample with *A* and *B* alleles, we will see three peaks in the frequency distribution centered at positions X_{norm} 0, 0.5 and 1.0, corresponding to SNP genotypes *AA*, *AB*, and *BB* (Figure 2).

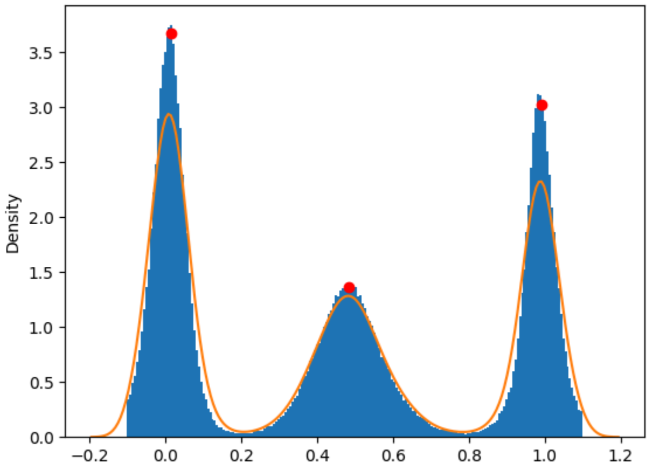

Figure 2. Histogram plot with density function and computed peaks according to the algorithm used for the AxioSAFE ploidy check analysis step. Note that three peaks are present. The sample in this example is the same one that was used in Figure 1.

For a triploid on the other hand, four peaks are expected, for SNP genotypes *AAA*, *AAB*, *ABB*, and *BBB*, centered at X_{norm} coordinates 0, 0.33, 0.66 and 1.0 (Figure 3).

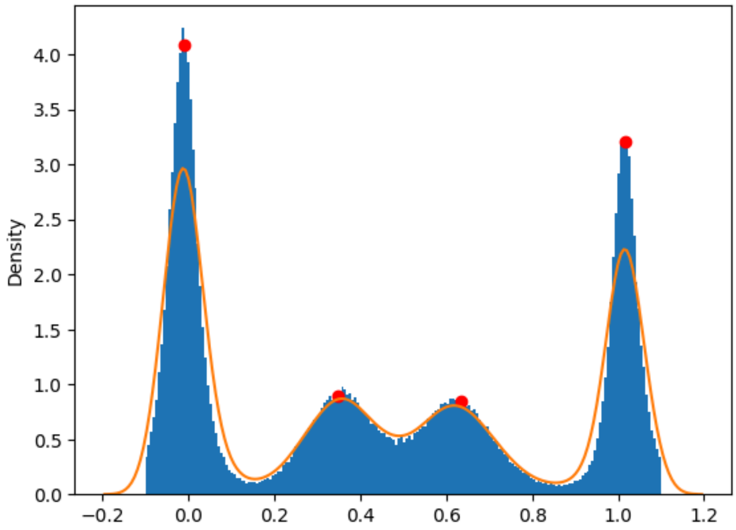

Figure 3. Histogram plot with density function and computed peaks according to the algorithm used for the AxioSAFE ploidy check analysis step. Note that four peaks are visible.

AxioSAFE `ploidy` checks the number of peaks to filter non-diploids. All samples where the number of peaks in the density function of the frequency distribution is above or below 3 are classified as non-diploid and labelled as 'NON_DIPLOID' in the MongoDB database. These samples will be excluded from all subsequent analysis steps.

When running on default settings, AxioSAFE `ploidy` will generate the ploidy frequency histogram plots (including the density function and peaks) for all samples identified as non-diploid, which are saved as images in the current working directory. This lets the user check the results immediately in a direct, visual way before moving on to the next step.

(2.3) Re-running AxAS

After running AxioSAFE `ploidy`, you may identify polyploid samples present within your panel. You may choose to ignore this and proceed with subsequent steps in the AxioSAFE pipeline: AxioSAFE will keep track of this and will not include polyploid samples in subsequent analysis steps.

However, these polyploid samples have contributed to the definition of the posterior genotype cluster definitions, possibly affecting the calling of diploid samples within the Axiom software. Thermo Fisher recommends excluding distinct ploidy levels from the genotyping analyses, requiring a new analysis on the trimmed panel. Therefore, you may alternatively choose to rerun AxAS in a new project with the raw .CEL files corresponding to samples of the target ploidy level only. The resulting data can then be fed back into AxioSAFE to continue on with the rest of the pipeline.

(3) SNP filtering

(3.1) Mendelelian errors and "Problematic SNPs"

Addressing Mendelelian-inconsistent and Mendelian-consistent errors is included in data curation for genotyping datasets. These errors are often encountered in downstream analyses (such as when examining the consistency of marker calls and phased haplotypes across known or potential parent-child relationships). They are usually caused by genotyping or phasing errors, which are usually related to biological events, limitations in the genotyping algorithm, and technological characteristics of the genotyping platform. As a result, they cannot fully be avoided at the experimental design level.

AxioSAFE is expected to reduce the proportion of errors by detecting and filtering out 'problematic SNPs'. These are SNPs in the dataset that cause a relatively high proportion of errors, and AxioSAFE `filterm` and `filterc` commands implement methods to identify them. SNPs that do not pass these filtering operations are labelled accordingly in the AxioSAFE database based on the specific filter they did not pass and are excluded when running subsequent commands or when exporting data at the end of a pipeline run.

The next section ('SNP cluster plot overview') first introduces the concept of the 'Axiom SNP cluster plot', a representation of the Axiom signal data space. Afterwards, the document provides examples for the SNP classes identified by AxioSAFE in the following section ('Problematic SNP examples and rationale'). Then, a third sub-section ('AxioSAFE SNP filter class examples') is dedicated to providing examples of altered data patterns in SNP cluster plots for each biological and technical cause.

(3.2) SNP cluster plot Overview

The Axiom signal data variables 'Contrast' and 'Size' (i.e., 'Log-Ratio' and 'Strength') constitute the major values used by the AxiomGT1 clustering algorithm implemented by the Axiom software programs (Axiom Analysis Suite (AxAS), and Analysis Power Tools, (APT)) to generate genotype calls. AxiomGT1 is applied to every SNP and assigns for each SNP a call to the samples available. The algorithm works based on pre-defined positions of the Axiom clusters corresponding to the three diploid genotypes *AA*, *AB,* and *BB*. It computes for each sample a confidence value which is used to judge whether the clustering result for that sample can be accepted (i.e., whether a sample belongs to a

cluster corresponding to a certain genotype). Samples that do not satisfy the internal thresholds for confidence are set as missing data ('NoCall' in the Axiom Genotype export file).

The cluster space can be viewed in the Axiom "SNP cluster plot", which can be accessed in AxAS (Cluster Plot tab). AxioSAFE also provides cluster plot visualization via the `review` command consistent with the format seen in AxAS. An example of a cluster plot as viewed in AxAS is shown in Figure 4.

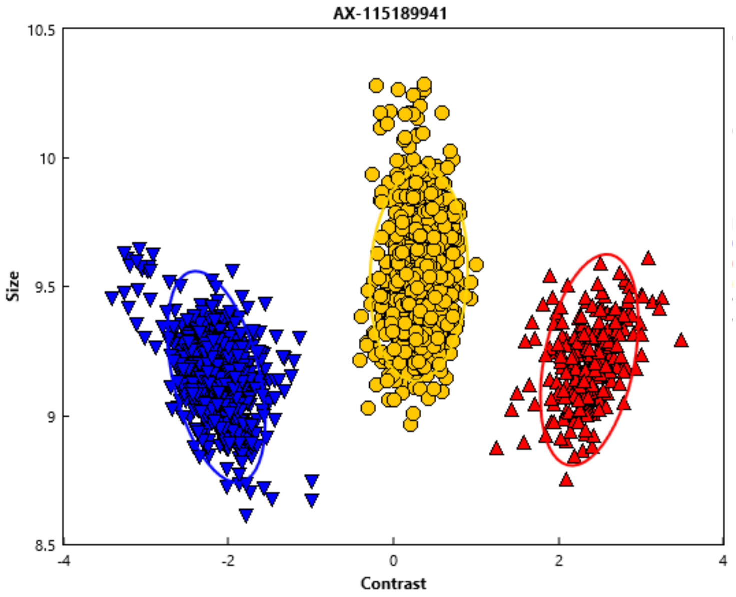

Figure 4. Axiom SNP cluster plot, standard case. Three clusters, AA, AB and BB are visible. Note that in large datasets with a lot of samples the likelihood of higher variation in the data pattern increases (as more samples make it possible to tell apart diversion from the standard case).

A generic SNP cluster plot will usually fall within a certain range of values, with the heterozygous cluster found around x-coordinate $x=0$ and the homozygous clusters being at an equal distance from the $x=0$ coordinate. The y-coordinates of the clusters will depend on the average intensity of the signal values, but usually falls in the 7-14 range, with ~6.7 corresponding to the y-coordinate for absence of signal based on the mathematical definition of 'Size' (see section 'Axiom Signal Data and True Raw Signal'). The clusters will be at relative positions that mimic a 'triangle' shape. This is what is called the **"standard case"** in this document: the best possible pattern that adheres to the pattern expected by the Axiom genotyping algorithm.

Studying cluster plots can help in identifying the full range of data patterns that can occur in real data, but the nature of the Axiom signal variables as transformations of the true signal measures (see section "Axiom Signal Data and True Raw Signal") adds a layer of variability to the final values. In true data various biological and technology-related aspects may affect SNP metrics as well, contributing to the overall variability of positions and sizes of the clusters, and the overall extensive space where samples can actually occur in the cluster space. For these reasons, the values are not always consistent with their counterparts in Infinium datasets, which means that **Infinium approaches are not always directly applicable to Axiom array data.**

Different cluster pattern cases are described below, including some example SNP cluster plot figures and possible explanations in terms of biology and technology.

(3.3) Problematic SNP examples and rationale

Additional Clusters

The standard case of Axiom SNP cluster plots includes only three clusters, corresponding to the Axiom genotypes in a diploid system (*AA*, *AB* and *BB*). However, in real genotyping data, a SNP may have sample data points cluster in such a way that more than three clusters can be identified when viewing the cluster plot manually.

The nature of these additional clusters varies depending on the cause behind them. Below some of the major cases are listed.

Polyploidy

Sample polyploidy leads to the appearance of additional clusters due to the wider spectrum of allele loads that are possible in polyploid settings (e.g., for a triploid sample we have four possible genotypes: *AAA*, *AAB*, *ABB* and *BBB*). Polyploid samples in diploid species are usually not common enough to lead to the appearance of clearly defined additional polyploid clusters; AxioSAFE assumes a diploid setting and the command `ploidy` identifies and filters out any polyploid samples from the current dataset.

Weaker or Stronger Alleles

In a standard case, a SNP cluster plot will show three genotype clusters corresponding to Axiom genotypes *AA*, *AB* and *BB*. The alleles *A* and *B* are assumed to have a comparable signal strength in the standard case, such that homozygous clusters *AA* and *BB* have roughly the same y-coordinate.

In real data, the appearance of additional alleles which correspond to a weaker signal intensity for the original *A* and *B* alleles. We can represent these as *a* and *b* (“small *A*”; “small *B*”) alleles, where allele *a* carries signal for the same channel as allele *A* but has lower signal intensity than allele *A*.

In practice, this leads to the appearance of additional clusters, as the presence of new alleles increases the number of possible genotype combinations. When weak and strong signal alleles are present, additional clusters are found close to or in-between the standard Axiom clusters because the ratio of signals is altered. Both cases can happen at the same time, which often results in complicated cluster plot cases with the appearance of 4, 5, or more clusters for a single SNP. Additionally, clusters may appear at lower size values as well, for instance, in the case of a homozygous lower-signal allele genotype (‘aa’ or ‘bb’). An example of a SNP featuring more than the three standard clusters is shown in Figure 5.

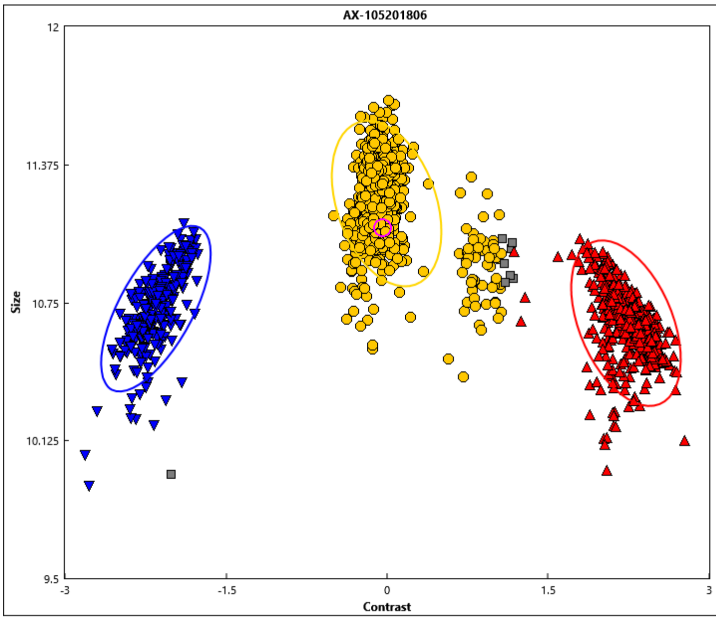

Figure 5. SNP cluster plot featuring multiple clusters, above the expected number of three clusters. This is a simple case where an additional cluster is present between the main Axiom genotype clusters AA and AB. The new cluster might for example be identified as Ab, although further analysis would be required.

Causes

The appearance of stronger or weaker allele signals is often related to problems in the binding mechanism of Axiom array probes and DNA fragments. Some of these include:

1. **Additional polymorphism(s) on a probe sequence:** the presence of an additional SNP near the target SNP in the genome sequence can result in weaker binding of the sequence to the Axiom probe, reducing the overall amount of signal. The strength of this effect depends on the position of the additional polymorphism, as SNPs close to the binding site will result in a much less stable binding site compared to SNPs close to the opposite side of the probe of 35bp.
2. **Secondary loci in the genome:** in some cases, especially for genomes that feature genome duplication in their evolution history (e.g., apple), a probe may have a secondary binding sequence in the genome. As a result, the Axiom probe will bind sequences from multiple loci for that SNP rather than just the target locus, resulting in a mix of signals from different genome coordinates. In the cluster plot, this can lead to both the appearance of multiple SNP clusters or background signal.

Null-Alleles

The presence of Null Alleles is another case that can lead to the appearance of multiple clusters. A “null allele” is the phenomenon where a ‘virtual’ allele exists that generates no signal for either the *A* or *B* channels (from this point on, represented using the ‘-’ character). If a null allele is present, we can have the appearance of additional clusters such as A-, B- and --, with the -- (Null) cluster in particular corresponding to the absence of signal intensity; in the case of no background signal, this roughly corresponds to Size value 6.7, based on the formulas for Size and Contrast. An example of a SNP featuring a possible “null allele” cluster is shown in Figure 6.

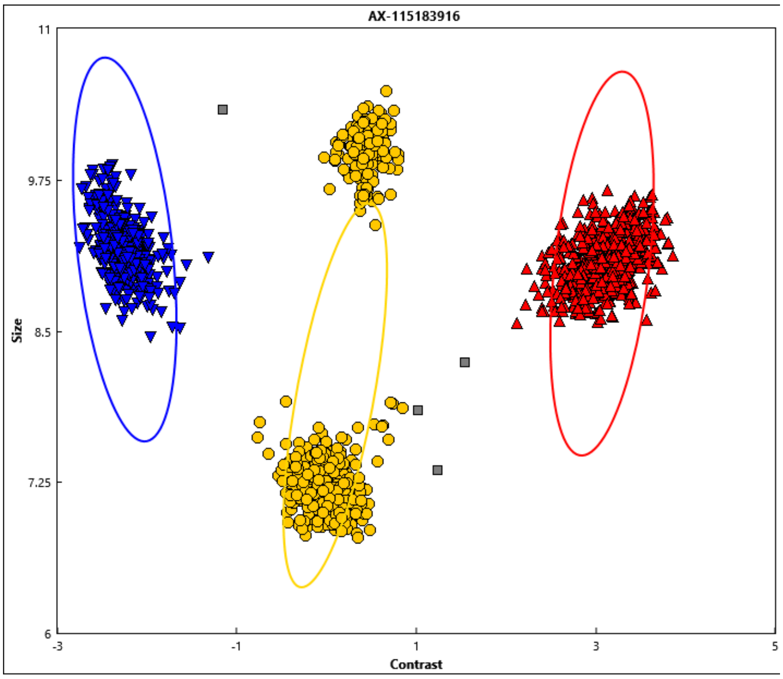

Figure 6. SNP cluster plot featuring a typical null allele loading. The position of the null allele cluster generally corresponds to the lowest signal intensity values for a specific cluster plot. The Axiom suite may classify the null allele as OTV, although this is not valid for all occurrences of null allele clusters.

Causes

1. **Additional polymorphism(s) on a probe sequence:** in some cases, the presence of an additional polymorphism on a probe sequence may lead to a complete failure of the probe rather than a weakening of the signal (such as for polymorphisms adjacent to the probe binding site, or when multiple polymorphisms are present on the probe sequence). This completely nullifies the allele signal, generating a null allele.
2. **Indels:** indels (insertion-deletions) are another type of genetic polymorphism that can occur on probe sequences. Their effect on a probe's functionality is often very strong, as an indel will cause a change in the offset of all base pairs before or after the polymorphism position, resulting in a much weaker binding strength that can lead to probe failure.

Background Signal

SNPs signal values may be affected by the presence of a weak, average or strong background signal that pushes signal values towards the A channel, B channel or both channels. This can be interpreted as general high values of signal strength, or as the presence of a fixed additional allele (e.g., in the presence of strong background signal for the *A* allele, the three Axiom genotypes *AA*, *AB* and *BB* become *AAAA*, *AAAB* and *AABB*). This will also notably cause an alteration of the cluster pattern (e.g., the cluster at the highest y-coordinate may not be the cluster corresponding to the heterozygous cluster for the target SNP). An example of a SNP featuring this particular cluster pattern is shown in Figure 7.

While AxioSAFE detects and labels most of the SNPs falling into this case, take into consideration that even in the presence of background signal a distribution with three separate clusters may be called correctly by the Axiom Analysis Suite. Further checks on the AxioSAFE SNP class "Unexpectedly Distributed Cluster SNPs" may therefore be needed to save SNPs.

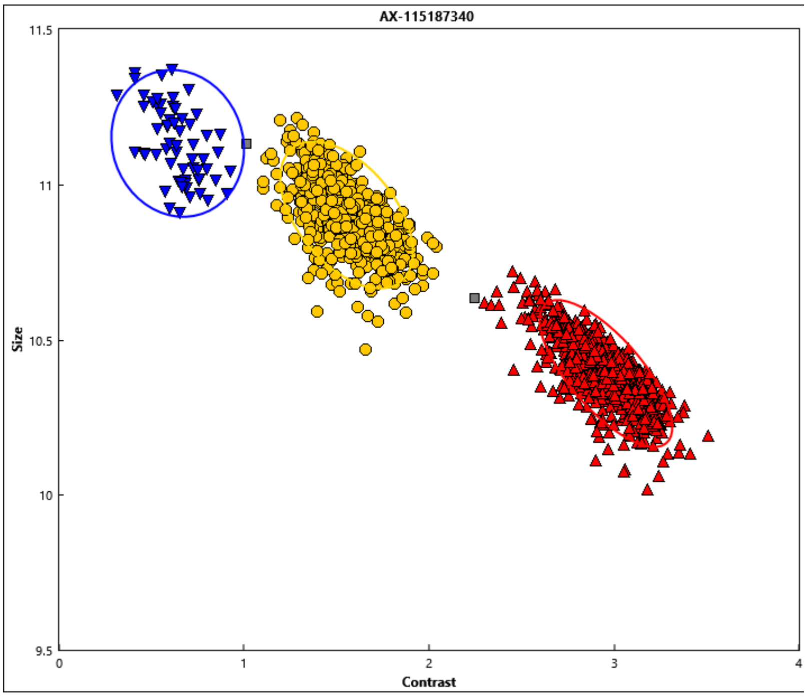

Figure 7. SNP cluster plot featuring a strong background signal case. For this SNP, we can interpret the cluster pattern as if a strong, constant AA signal is present for all samples: this means that the standard genotype clusters BB, AB and AA correspond to allele loads AABB, AAAB and AAAA instead. The high values of Size are also a clue towards the presence of background signal. Take into account that despite the non-standard distribution, the actual genotype calls remain correct when it comes to the actual polymorphism.

Causes

1. **Secondary locus with a constant signal strength:** when a secondary locus for the SNP's probe is present in the genome, the most common effect is the presence of background signal for a fixed signal allele (if that locus corresponds to a position in the genome without polymorphisms). Either 'A' or 'B' signal channel will be read for all samples on top of the true SNP signal, altering all final signal values accordingly as the secondary locus competes with the target locus for the probes present on the array plate.

Homozygous-Homozygous Clusters

A recurring case of non-standard cluster distributions in Axiom data is the 'homozygous-homozygous' case, where only the two homozygous clusters are present while the heterozygous cluster seems completely or almost completely absent. This case occurs when the Axiom Analysis Suite's genotype calling algorithm does not correctly interpret the presence of only two clusters in the SNP cluster plot.

This case covers a wide variety of problematic SNPs. A common occurrence happens when a single homozygous and the heterozygous clusters are present, and AxAS calls them both as homozygous. However, different cases may happen that lead to the appearance of only two clusters, such as when the heterozygous cluster and one of the two homozygous clusters become merged with each other. Two SNPs are shown in Figure 8as examples of homozygous-homozygous clustering patterns.

The current version of the AxioSAFE pipeline filters out all 'homozygous-homozygous clusters' SNPs. Whether each of these SNPs may be saved requires further analysis in order to identify the true

genotype that may be found within the samples for every given SNP, with methods such as alignment tools with reference genomes, tests with known mapping populations when available in the panel, and checking the overall signal intensity.

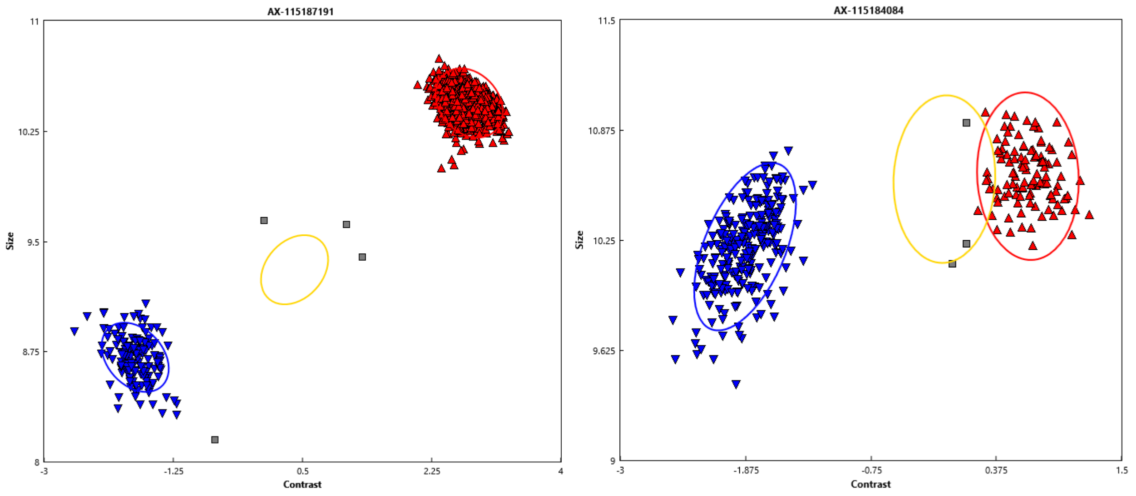

Figure 8. SNP cluster plots with a typical “Homozygous-Homozygous Clusters” example, two separate examples. For this case the two SNPs come from Axiom Analysis Suite projects with different panel sizes: the full Axiom 480K from (Bianco et al, 2016), and a subset of 4 array plates. Different panel sizes can result in different cluster plot patterns, further complicating the cases studied when looking at SNP cluster plots.

Angled Clusters

Due to the nature of Axiom genotyping and signal data, standard SNP cluster plot cases sometimes feature clusters of samples that are ‘sloped’: the sample points appear to cluster along an angled axis rather than being along an axis parallel to the y-axis. For low values, the slope of Axiom clusters and their close proximity can result in partially overlapping clusters and, therefore, poor clustering.

For high signal values, SNPs will fall on the other hand in a different special case, where the angle of the slope becomes more noticeable and SNP clusters become clearly compressed along the cluster’s axis. An example of a SNP falling into this case is shown in Figure 9. The current version of AxioSAFE does not capture these SNPs, since no saturation filter based on overall signal intensity is present, which may be taken into account in future versions of the pipeline.

Causes

1. **Limitations of high signal values in Axiom data:** AxAS has functionalities for viewing the B to A signal intensity plots. By viewing this plot, we can take note of the fact that the high signal values found for sloped cluster SNP cluster plots do not go beyond a maximum (the exact value of which varies; most high signal values are found around the 3800-4400 range). An example is provided in Figure 10, using the SNPs shown in Figure 4 and Figure 9 for comparison purposes. This gives the impression that values are ‘cut-off’ at the end of the space covered by Axiom Signal plots, which plays a role in the final “compressed cluster” pattern that we see for the SNPs. This virtual ‘cut-off’ is an inner feature of the Axiom platform that is caused by transformations applied to the signal values outputted by the genotyping machine, rather than a fixed cut-off thresholds, although we see signal data points

being limited and compressed as soon as signal values for both the A and B channel go above 3800. Further investigation on the nature of this aspect of Axiom data is needed.

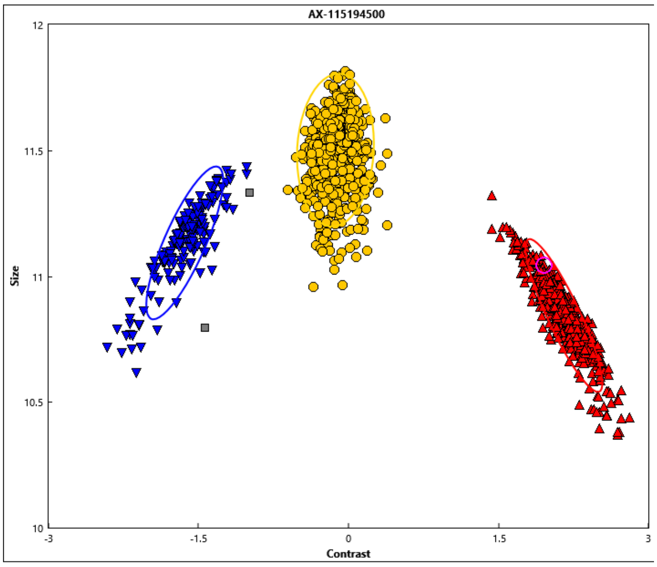

Figure 9. SNP cluster plot, sloped clusters in the case of very high values. Homozygous clusters AA and BB appear visibly compressed along the direction of the slope.

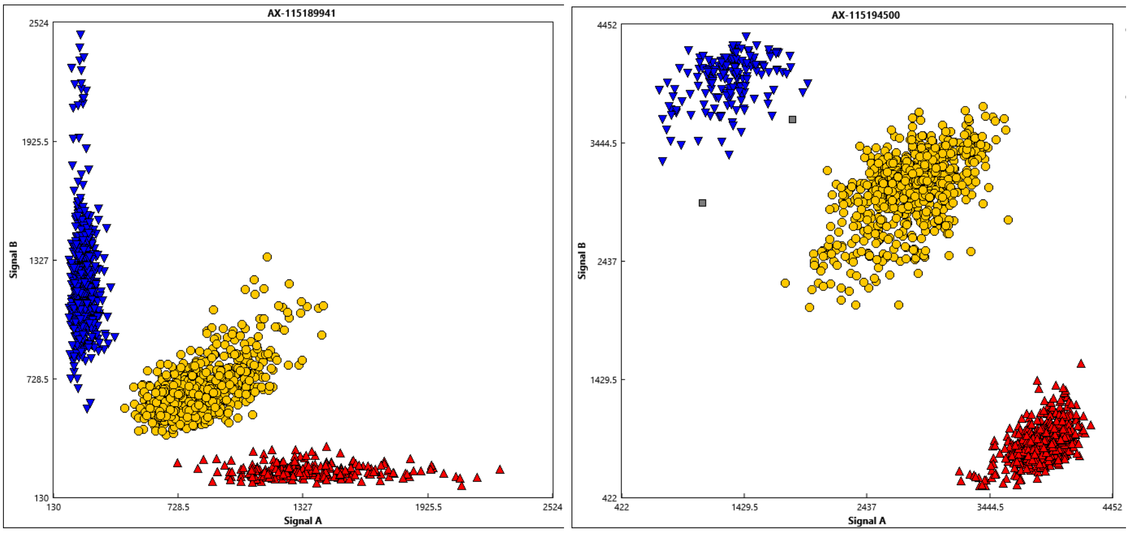

Figure 10. Sloped cluster case, comparison of original Signal value plot as seen in the Axiom Analysis Suite. The signal A and B value plotted here correspond to the A and B signal (after scaling transformation, but before logarithm transformation, see section 'Axiom Signal Values and True Raw Signal'). The plot on the left is the same standard case SNP in Figure 4 that was picked as an example figure in the 'SNP Cluster Plot Overview' section. The plot on the right has sloped clusters that are compressed rather than spread out, with high intensities that partly exceed the 3800 threshold for channel saturation, which is where the additional assessments are used to estimate intensities. Their regression lines also do not converge towards the same point of the heterozygous clusters, as seen in the plot on the left.

Additional Factors for problematic SNPs

Array Plate Bias

When working with multiple array plates, plate bias may cause the appearance of alterations to the typical cluster plot pattern. Array plate biases are usually detectable a priori by highlighting the sample set by array plate (possible in the Axiom Analysis Suite SNP cluster plot view), as such an effect will usually affect all samples belonging to the same plate, while excluding the rest. The most recent version of AxAS has options to account for multi-plate analysis, but a check on the presence of biases should still be included for data curation purposes.

Copy Number Variations

Copy Number Variations (CNV) consist of a change in the number of copies of a section of the genome across different individuals. In the context of an SNP array genotyping analysis, CNVs can lead to changes in the amount of signal read by a specific SNP in the array, as the quantity of DNA present that covers that specific locus may increase due to repetition.

Effective DNA Quantity

Another factor that plays a role in the variability in SNP metrics among samples is the quantity of available nuclear DNA. When running SNP-arrays, efforts are made to use equal amounts of DNA for the different samples. However, the overall quantity of DNA extracted from a sample will not be limited to nuclear DNA, but may also include organelle DNA (i.e., mitochondrial DNA and chloroplast DNA). Their relative contribution is affected by the physiological stage of the tissue: for example in apple, just appearing leaf buds will have a lower proportion of chloroplast DNA than unfolded leaves, thus causing variation in the quantity of effectively available nuclear DNA. Such variation may affect the accuracy of SNP calls, especially in having sub-clusters close to each other (e.g., clusters with low signal alleles) separated well among samples from different harvest periods.

Complex SNPs: multiple factors may be involved in for a single polymorphism

A lot of factors, cases and biological events described in this document are not mutually exclusive. Some may apply to the same SNP in parallel to each other, while others may affect a SNP to varying degrees. In practice, what we see in real data is a **mixture of complex 'problematic SNP' cases with a cluster pattern, position and number altered in multiple ways,** which are not necessarily easy to study and requires careful consideration before assigning a specific cause to that cluster pattern. Further analysis that makes use, for instance, of mapping populations parental relationships, or cross-references the SNP calls with the WGS data available for the given species, may be needed.

Figure 11 provides an example of a SNPs where, possibly, multiple factors are involved in defining the final cluster pattern that we see in the SNP cluster plot.

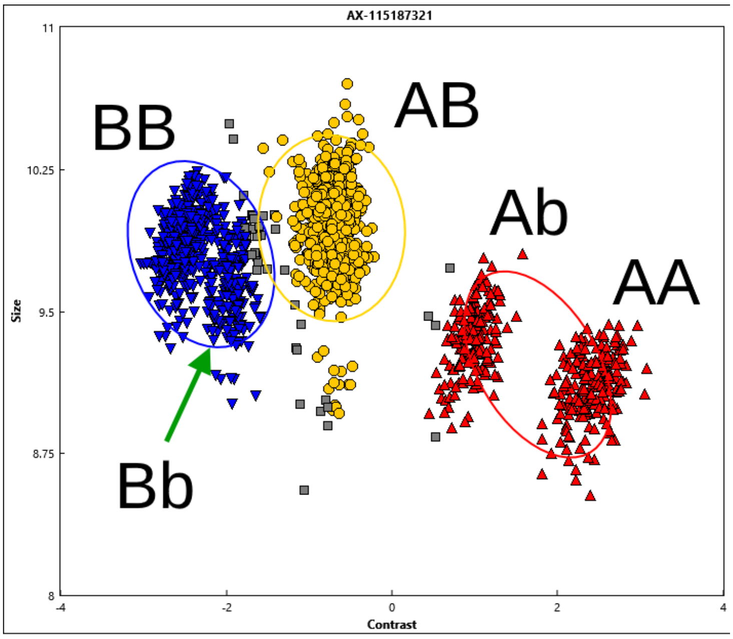

Figure 11. SNP cluster plot featuring multiple clusters, above the expected number of three. Notice that in this case we have 5 clusters in total, with a good separation for the clusters on the right, while the clusters on the left are partially merged. This example can tentatively be explained by adding two additional low-intensity alleles ‘a’ and ‘b’. The allele loads for the clusters in the plot, from the left, would be ‘BB’, ‘Bb’, ‘AB’, ‘Ab’, ‘AA’.

(3.4) AxioSAFE SNP filter class examples

AxioSAFE SNP classes do not correspond 1:1 to the SNP cases described in the previous section, which the curation pipeline provides partial coverage for. This sub-section includes a list of SNP classes identified by the curation pipeline, with examples for each SNP class label that is covered and assigned by AxioSAFE’s SNP filtering commands.

(1) **‘Single Cluster SNPs’**

SNPs that display only one genotype cluster in the SNP cluster plot.

MongoDB label: “MONO_HIGH_RESOLUTION”. Corresponds to the “MonoHighResolution” ‘ConversionType’ category in AxAS.

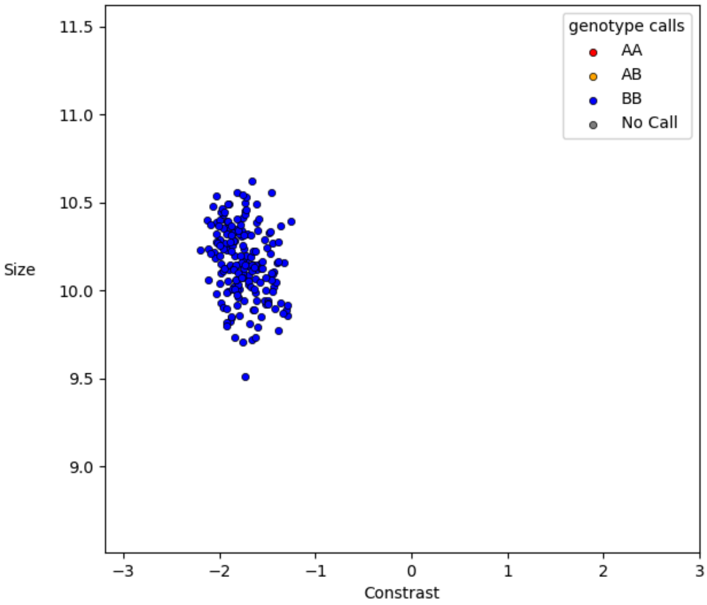

(2) **‘Homozygous-Homozygous cluster SNPs’ -** SNPs showing a classification of of two homozygous, with an absent or extremely small heterozygous cluster; SNPs are assigned to this class based on the counts of genotype calls of the three expected Axiom clusters.
MongoDB label: “HOM_HOM_CLUSTERIZATION”

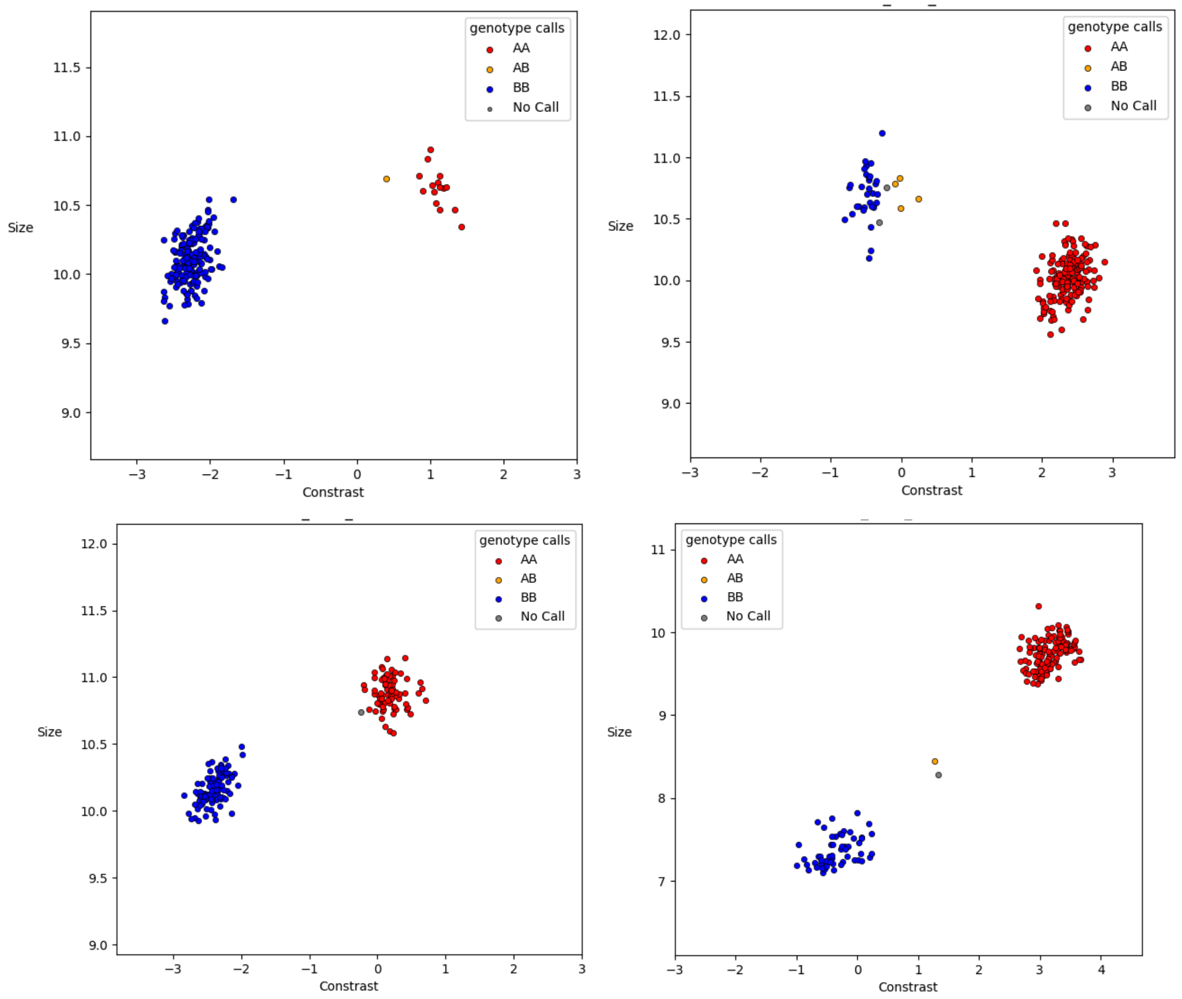

(3) **‘Unexpectedly Distributed Cluster SNPs’-** SNPs with genotype clusters whose x-axis coordinates fall in unexpected (i.e., non-standard) positions; identified by computing the coordinate of the midpoint between the coordinates of the two homozygous clusters, and checking its distance from coordinate 0. This category mostly covers SNPs with strong background signal that pushes Axiom clusters towards the left or the right.
MongoDB label: “UDC_CLUSTER_DISTRIBUTION”.

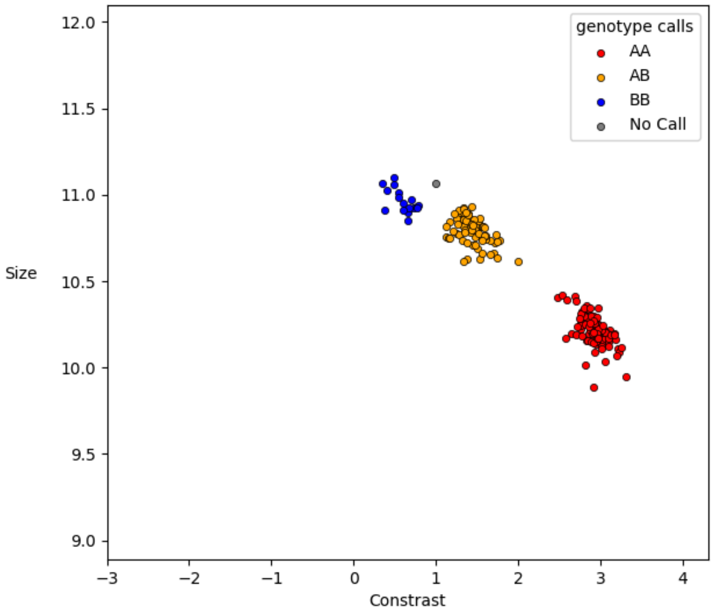

(4) **‘Axiom-metrics-threshold SNP’** - SNPs flagged based on their value(s) for Axiom metrics “Call Rate”, “FLD”, “HomFLD”, “HetSO”, “HomRO”, or cluster variance values/variance “z-scores” values. The “Call Rate” filter covers the SNP QC on call-rate filter implemented by AxAS, while all the filters on the other SNP metrics cover additional AxAS filters implemented via the “Reanalyze–>Regenrate SNP metrics” functionality in the probeset view. For each Axiom metric, AxioSAFE tests a separate filter and assigns a distinct label, so SNPs in this class are assigned multiple labels (‘sub-classes’).
MongoDB labels are reported for each sub-class:

Call Rate (“CALL_RATE_AXIOSAFE”). Low quality SNPs.

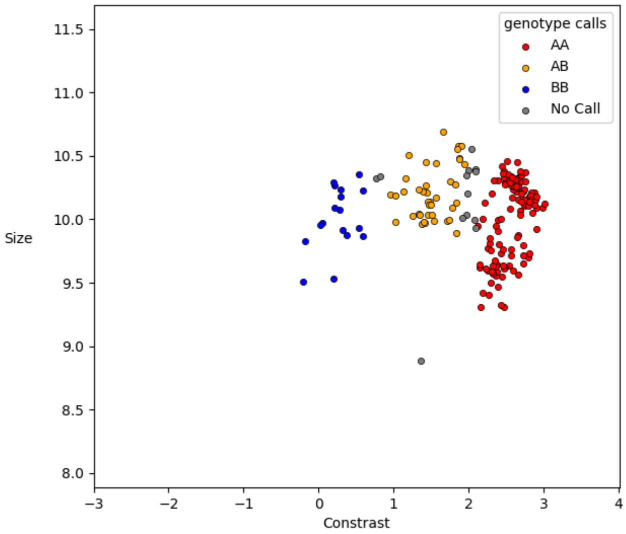

FLD (“FLD_MAIN_VALUE_RANGE”). SNPs with poor clustering.

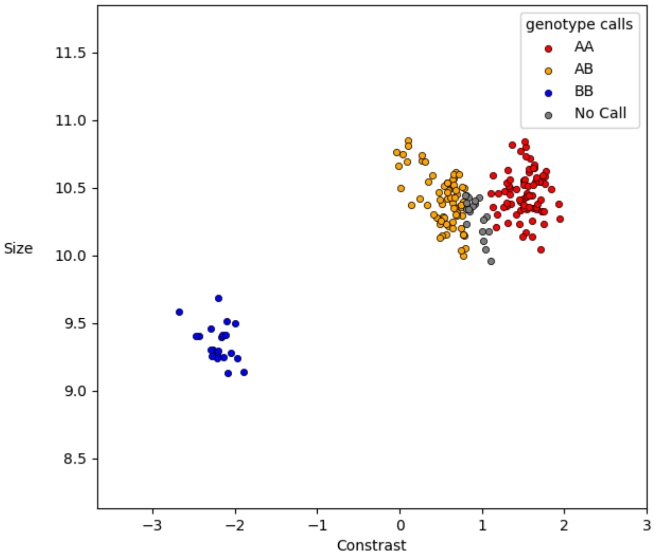

HomFLD (“FLD_HOMOZYGOUS_VALUE_RANGE”). SNPs with poor clustering; this filter covers cases where only the homozygous clusters are affected by the poor clustering.

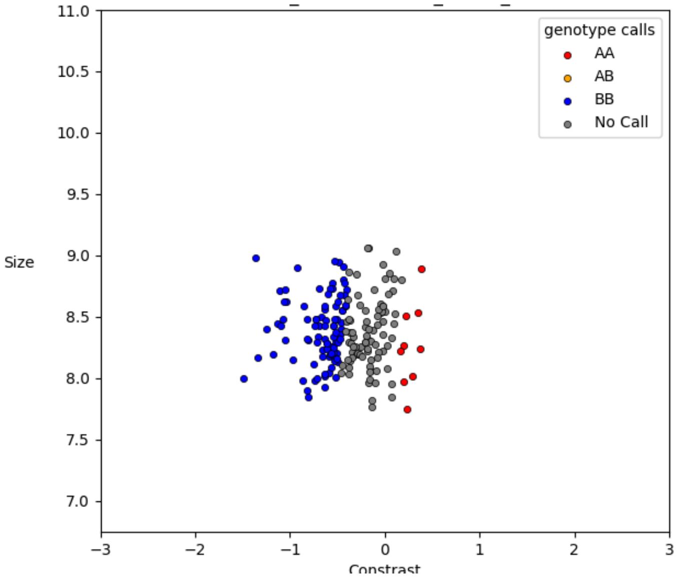

Heterozygous Strength Offset - HetSO (“HETSO_FILTER”). Filter on the “HetSO” SNP metric value. Captures SNPs with a large covered value range on the y-axis.

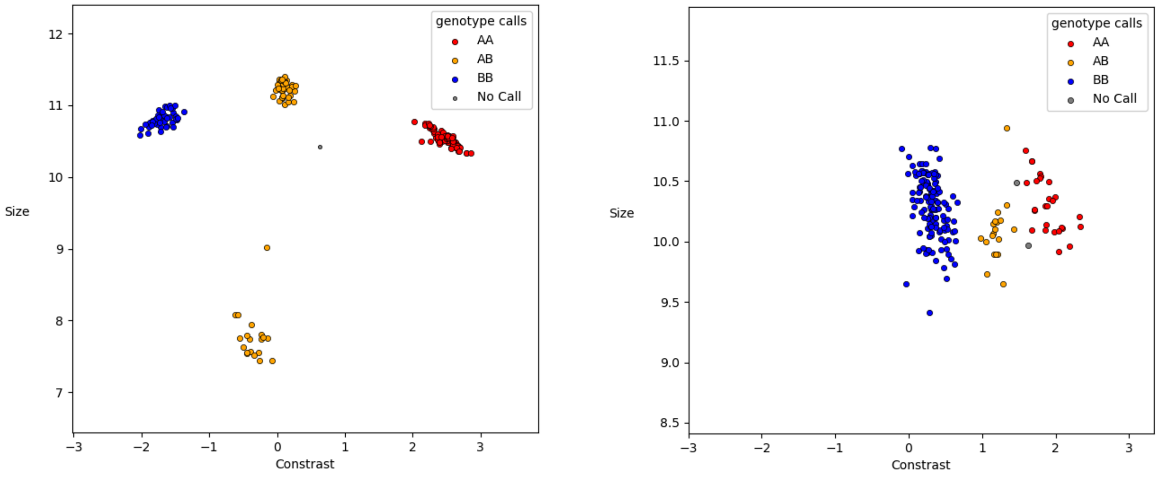

Homozygous Ratio Offset - HomRO (“HOMRO_FILTER”). Filter on the “HomRO” SNP metric value. Captures SNPs with some non-standard cluster patterns that result in unexpected distributions on the x-axis.

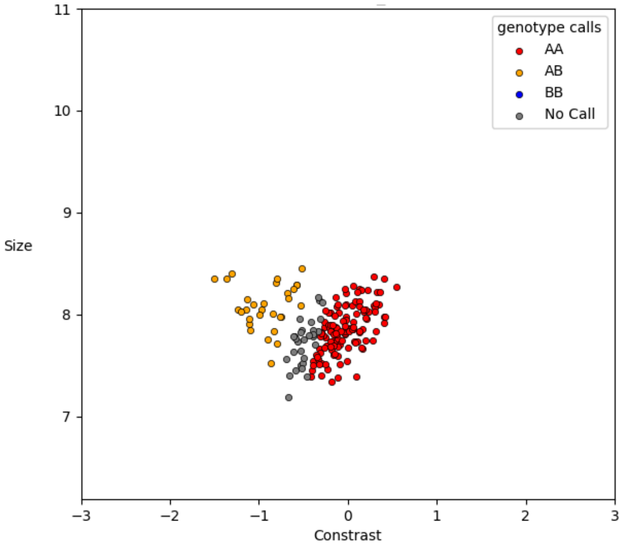

Filter on Variance (“VARIANCE_FILTER”). Based on configuration file parameters, this filter can either be a filter on the variance values, or the variance-z-score values, for each SNP Axiom cluster and dimension. This filter captures SNPs that feature genotype clusters with high variances, often caused by a larger than expected spread of the points assigned to a certain Axiom cluster.

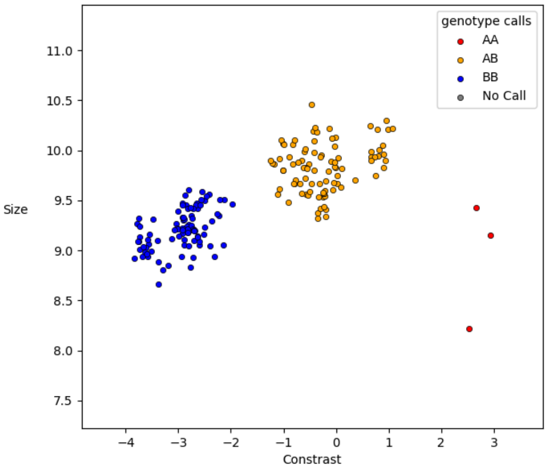

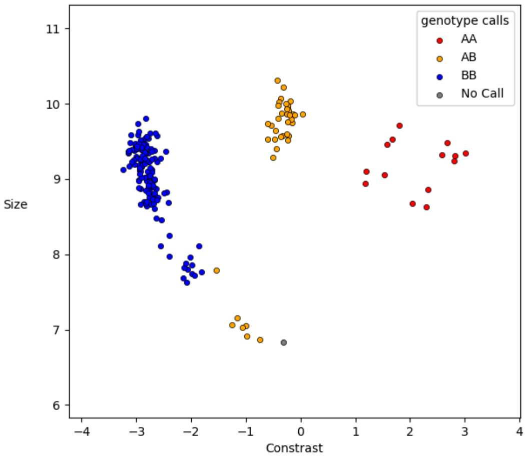

(5) **‘Multiple Cluster SNPs’** - SNPs exhibiting more than the three Axiom standard genotype clusters; SNPs in this class are identified through the sub-clustering algorithm of the ‘filterc’ command.
MongoDB label: “SUBCLUSTERING FILTER”. See section “Sub-Clustering Algorithm”.

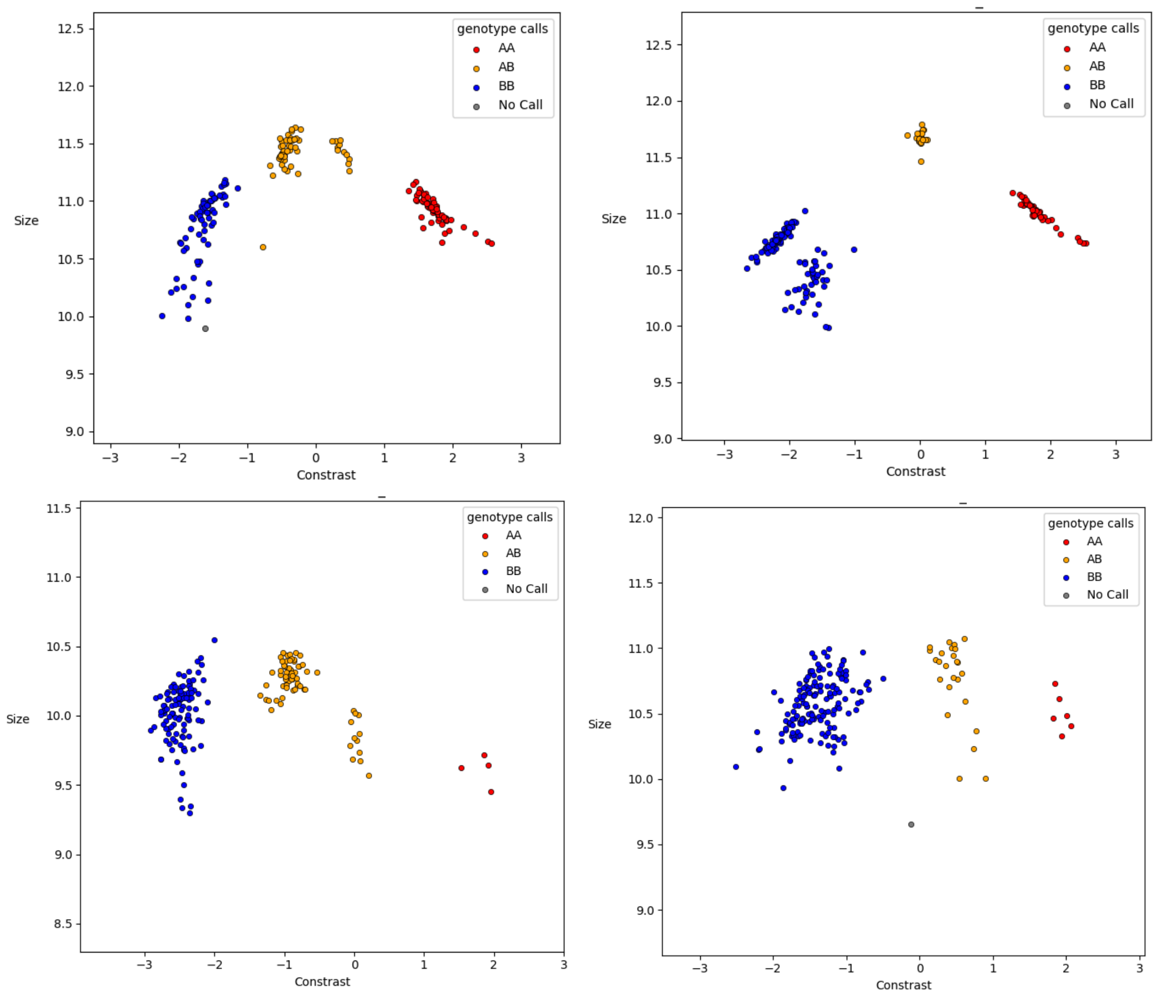
genotype calls
AA
AB
BB
No Call
Size
Constrast

(3.5) Filter Clusters: Sub-clustering Algorithm

The AxioSAFE `filterc` command implements an algorithm (from this point on "subclustering algorithm") that uses a simplified method for identification of subclusters within Axiom clusters.

The rationale is as follows: in the presence of more than three clusters or clusters close to each other, the AxiomGT1 genotyping algorithm will often group clusters into one of the Axiom standard clusters (AA, AB, BB).

AxioSAFE's `filterc` leverages this aspect to simplify the problem of the identification of multiple clusters (avoiding the need for an alternative clustering algorithm to AxiomGT1 to be applied on the entire data series for each SNP). Rather than reclustering the entire dataset, `filterc` implements the following workflow:

1. Implement a preliminary filter in order to select only SNPs that are likely to feature multiple clusters, based on 'cluster ranges' (the minimum space covered by Axiom clusters on x-axis and y-axis).
2. For every SNP in the resulting working dataset:
    a. Select an Axiom cluster.
    b. For all samples called for that cluster, perform k-means clustering based on the provided parameters (by default, only k-means with k=2 is performed; different values of k or tests over multiple values of k can be set via the configuration file).
    c. Compute quality metrics 'Mean Silhouette Coefficient' and 'Davis-Bouldin Index', and test them against the thresholds (default conditions: MSS > 0.6, DBI < 0.4).
3. If at least one of the quality metrics satisfies the conditions, then `filterc` assumes that a subcluster occurrence was found. The SNP is labelled as 'SUBCLUSTERING_FILTER' (i.e. filtered out), and the program moves to the next SNP.
4. If none of the quality metrics satisfies the condition, then `filterc` assumes that no subclusters are present for the current Axiom cluster, and moves on to the nextAxiom cluster.
5. If no Axiom cluster is left, `filterc` assumes that the SNP does not feature subclusters at all. The SNP is not labelled for filtering, and `filterc` moves on to the next SNP.
6. Loop until all SNP in the working dataset are processed.

(3.6) AxioSAFE filters overview

AxioSAFE's filtering operations have been referenced in the previous section. The table below provides an overview of all SNP classes and labels available in AxioSAFE

AxioSAFE filter and SNP class label	AxAS counterpart	Implementation	Data patterns identified	Causes
Mono High Resolution `MONO_HIGH_RESOLUTION`	ConversionType label 'Mono High Resolution'	**filterm** Count of genotype calls.	Single clusters.	Non-informative SNPs (locus has no polymorphism in the studied germplasm)
Homozygous-Homozygous Clusters `HOM_HOM_CLUSTERIZATION`	/	**filterm** Count of genotype calls.	Two homozygous clusters shown in the SNP cluster plot.	Misinterpretation of cluster plots by the AxAS Genotype calling algorithm
Unexpectedly Distributed Clusters `UDC_CLUSTER_DISTRIBUTION`	/	**filterm** abs(midpoint between homozygous cluster coordinates) should be above the threshold.	Non-standard cluster distribution on x-axis (how well centred around coordinate 0 the clusters are on the x-axis)	Unexpected sources of signal intensity (e.g., background signal).
Fisher Linear Discriminant `FLD_MAIN_VALUE_RANGE` `FLD_HOMOZYGOUS_VALUE_RANGE`	ps-metrics thresholds	**filterm** threshold on Axiom metrics.	General quality of Axiom clustering.	All non-standard patterns that lead to poor clustering.
Heterozygous Strength Offset `HETSO_FILTER`	ps-metrics thresholds	**filterm** threshold on Axiom metrics.	Non-standard cluster distribution on y-axis.	Additional clusters for the heterozygous cluster, merged additional clusters
Homozygous Ratio Offset `HOMRO_FILTER`	ps-metrics thresholds	**filterm** threshold on Axiom metrics.	Non-standard cluster distribution on x-axis.	Unexpected sources of signal intensity
Variance Filters `VARIANCE_FILTER`	ps-supplemental thresholds	**filterm** threshold on Axiom metrics.	Larger than expected x-axis and y-axis range covered by Axiom clusters.	Merged additional clusters, multiple cluster called in a single Axiom cluster
Call Rate `CALL_RATE_AXIOSAFE`	SNP QC call-rate	**filterm** threshold on Axiom metrics.	Generic filter on low quality SNPs, useful when choosing to use all SNPs from the array.	Failing SNP, poor SNP quality.
Multiple Clusters `SUBCLUSTERING_FILTER`	/	**filterc** subclustering algorithm.	Presence of multiple clusters.	Multiple causes, e.g., presence of null-alleles and suballeles.
Mendel Error Filter `MENDELIAN_ERROR_FILTER`	/	**filterp** Based on the Mendel error report from 'pedgr'.	Multiple non-standard patterns not identified or missed by previous steps.	Other genotyping errors not captured by previous steps.
Manually picked SNPs `MANUAL_FILTER`	/	**review** Option to manually set SNP class labels to this	Any data pattern identified visually by the user as problematic via "review".	/

		class		

References

Axiom Software Documentation: Provided information as reported in this document covering Axiom signal data transformations are available in the "Axiom Genotyping Solution Analysis Guide", direct link https://assets.thermofisher.com/TFS-Assets%2FLSG%2Fmanuals%2FMAN0018363-AxiomDataAnalysis-UG-RUO.pdf, found at the Thermo Fisher "Microarray Analysis for Population Genomics" reference website: "https://www.thermofisher.com/it/en/home/life-science/microarray-analysis/applications/predictive-genomics/population-genomics/software.html".

RefereChagné,D. *et al.* (2015) Polyploid and aneuploid detection in apple using a single nucleotide polymorphism array. *Tree Genet. Genomes*, **11**, 94.

Howard,N.P. *et al.* (2023) Pedigree reconstruction for triploid apple cultivars using single nucleotide polymorphism array data. *PLANTS PEOPLE PLANET*, **5**, 98–111.

Peiffer,D.A. *et al.* (2006) High-resolution genomic profiling of chromosomal aberrations using Infinium whole-genome genotyping. *Genome Res.*, **16**, 1136–1148.

Vanderzande,S. *et al.* (2019) High-quality, genome-wide SNP genotypic data for pedigreed germplasm of the diploid outbreeding species apple, peach, and sweet cherry through a common workflow. *PLOS ONE*, **14**, e0210928.
